# Construction of a high density genetic map of an interspecific cross of *Capsicum chinense* and *Capsicum annuum* and QTL analysis of floral traits

**DOI:** 10.1038/s41598-018-38370-0

**Published:** 2019-01-31

**Authors:** Zhangsheng Zhu, Binmei Sun, Jianlang Wei, Wen Cai, Zhubin Huang, Changming Chen, Bihao Cao, Guoju Chen, Jianjun Lei

**Affiliations:** Key Laboratory of Horticultural Crop Biology and Germplasm innovation in South China, College of Horticulture, South China Agricultural University, Guangzhou, 510642 China

## Abstract

The yield of pepper plants (*Capsicum* spp.) is their most important trait and is affected by the flower number and flowering time. *Capsicum annuum* produces a single flower per node and has an early flowering habit. By contrast, *Capsicum chinense* yields multiple flowers per node and has a late flowering character. However, the genetic mechanism underlying the control of these floral traits remains largely unknown. In this study, 150 F_2_ populations from an interspecific cross between the inbred lines 740 (*C*. *chinense*) and CA1 (*C*. *annuum*) and their parents were used to construct a molecular genetic linkage map using the specific length amplified fragment sequencing (SLAF-seq) technique. This linkage map, spanning 1,586.78 cM in length, contained 9,038 markers on 12 chromosomes, with a mean marker distance of 0.18 cM. Phenotypic data on the flowering time and flower number per node were collected over multiple years, and QTL analysis identified 6 QTLs for the flowering time and flower number per node by composite interval mapping (CIM) and genome-wide composite interval mapping (GCIM) methods at least in two environments. The candidate genes within the major QTL were predicted. In the major flowering time QTL, the candidate gene *Capana0*2*g000700*, which encodes the homeotic protein APETALA2, was identified. Quantitative reverse-transcription PCR (qRT-PCR) analysis indicated that its expression level in 740 was higher than that in CA1. Gene expression analysis indicated that the expression of *Capana02g000700* was significantly upregulated in flowers, and many floral development-related genes were found to be coexpressed with *Capana02g000700*, supporting the function of this gene in association with flowering time in *C*. *chinense* and *C*. *annuum* species.

## Introduction

Pepper (*Capsicum* spp.) is an economically important plant of the Solanaceae family, whose fruits are consumed as vegetables and food additives for their unique color, pungency, and aroma in many regions of the world, particularly in Asia and South and Central America^1,2^. Peppers are also grown for use as an ornament, for chemical industries, and for their pain-killing and medicinal properties^3^. Approximately 35 species of *Capsicum* are native to Central America^4^, of which *Capsicum annuum*, *Capsicum chinense*, *Capsicum frutescens*, *Capsicum baccatum*, and *Capsicum pubescens* have been domesticated and are now cultivated in different parts of the world^5^. Of these species, *C*. *annuum* is the one most widely grown, and its yield accounts of for 80% of pepper fruit production worldwide^6^. However, it is low in disease resistance and adaption to the humid lowland tropics, where, at least in Latin America, it has been replaced by *C*. *chinense* and *C*. *frutescens*^6^. Among the five domesticated *Capsicum* species, enormous variation were observed in plant architecture, flower-, leaf-, fruit-, metabolism- and disease resistance- related traits^7,8^. The yield of pepper is the most important trait, affected by factors such as the flower number and flowering time. Previous studies have strongly indicated that selection for more flowers and moderately early flowering can substantially enhance yields in various horticultural types of pepper^6^. *C*. *annuum* has one flower per node and early maturity, whereas *C*. *chinense* plants have multiple flowers (always two to four) per node and a late flowering time habit. Therefore, interspecific hybridization of *C*. *annuum* with *C*. *chinense*, with the multiple flower trait being transferred into *C*. *annuum*, may be potentially useful to increase the yield and enhance uniform maturity, which may make mechanical harvesting feasible^9,10^. However, transferring superior traits from *C*. *chinense* into *C*. *annuum* to develop viable commercial varieties is very time-consuming and expensive because of the number of backcrosses required. Since most of the traits mentioned above are quantitatively inherited or controlled by multiple major genes^1,9,11^, the discovery of the QTLs or major genes that govern these traits in various backgrounds is imperative; furthermore, the use of molecular-assisted selection can shorten breeding cycle and accelerate the breeding process of new varieties of pepper. During the past few decades, many genetic maps, including integrated maps, have been constructed for peppers using either intraspecific^12,13^ or interspecific^8,14,15^ populations to identify the QTLs of horticultural traits^16^. However, most of these genetic maps were low-density, and many identified QTLs covering large region lead hard to use for molecular assistant selection.

A genetic map, especially a high-density genetic map, provides an important foundation for QTL mapping and major QTL cloning. In *Capsicum* species, a number of interspecific and intraspecific genetic maps have been constructed by using conventional methods in previous studies^1,15^. However, the current number of markers is too small to build a high-density genetic map, which limits the efficiency and accuracy of QTL mapping. Next-generation sequencing (NGS) technologies can be used to detect large quantities of SNP markers in the whole genome for high-resolution genetic map construction. Several methods combine NGS with restriction enzyme digestion to reduce the complexity of the target genomes, including genotyping-by-sequencing (GBS-seq)^17^ and restriction site-associated DNA sequencing (RAD-seq)^18^. The selection of digested DNA fragment sizes is critically important to improve the efficiency of tag utilization. Unlike GBS-seq, which does not select the size of the digested fragment before PCR amplification, the RAD-seq conducts the size-selection step of the digested fragment before PCR amplification^19^. However, traditional RAD-seq technology has several shortcomings, such as more operation steps and shorter read length. By combining bioinformatics and RAD-seq technology, specific-locus amplified fragment sequencing (SLAF-seq) is developed^20^. SLAF-seq applies a bioinformatics approach to simulate the results of enzyme digestion, selects the most suitable restriction enzymes for double digestion, and then sequences the PCR-amplified fragments on an Illumina sequencer. This approach can effectively avoid repetitive sequences in the genome, develop SNP markers with uniform distribution in the genome, increase the effective reads obtained by sequencing, and improve the efficiency of molecular marker development^20^.

To date, many pepper intraspecific high-density genetic maps have been reported^21–23^. However, interspecific high-resolution genetic maps have rarely been reported in peppers^24^. Due to an abundance of polymorphic DNA sequences in interspecific individuals, the construction of a high-density genetic map based on SNPs markers of *Capsicum* species is possible. In this study, based on SLAF-seq, we constructed a high-density genetic map of an interspecific cross of *C*. *annuum* and *C*. *chinense*. Furthermore, we used a high-density genetic linkage map to detect QTLs for certain traits of peppers: flowering time and flower number per node.

## Results

### SLAF sequencing and genotyping of the interspecific cross F_2_ population

In this study, the 150 interspecific cross F_2_ populations and their parents were genotyped using SLAF-seq technology. Based on the results of the SLAF pilot experiment, *Hae*III was used for SLAF library construction. The library comprised SLAF fragments ranging from 414 to 514 bp in length. After high-throughput sequencing of the SLAF library, 183.83 Gb of raw data was generated. In total, 749.26 M pair-end reads were obtained for both parents and 150 progenies, with an average of 4.92 M reads for each individual line. For quality control processing, reads of low-quality were discarded during quality checks in each cycle. This dynamic process was repeated until the average genotype quality score of all SLAFs reached the cut-off value of 30 (quality scores of at least 30, indicating a 1% likelihood of an error and thus 99.99% confidence). On average, Q30 was 92.78%, and the GC content was 38.21%. After reads clustering, 406,563 high-quality SLAFs were detected. The average depths of these SLAFs were 90.34 (male parent) and 58.61 (female parent) for parents and 11.74 for each individual progeny (Table 1).

**Table 1:** SLAF-seq data summary for *Capsicum* interspecific F_2_ population.

Samples	Total Read	Total Bases	Q30 percentage (%)	SLAF Number	Total Depth	Average Depth (×)
CA1	34,204,510	8,410,520,418	91.65	267,276	24,146,326	90.34
740	23,641,770	5,812,970,664	90.83	208,177	12,201,135	58.61
offspring	4,609,428	1,130,764,269	92.8	249,183	2,925,009	11.74
Total	749,260,524	183,838,131,504	92.78	406,563		

**Figure 1:**
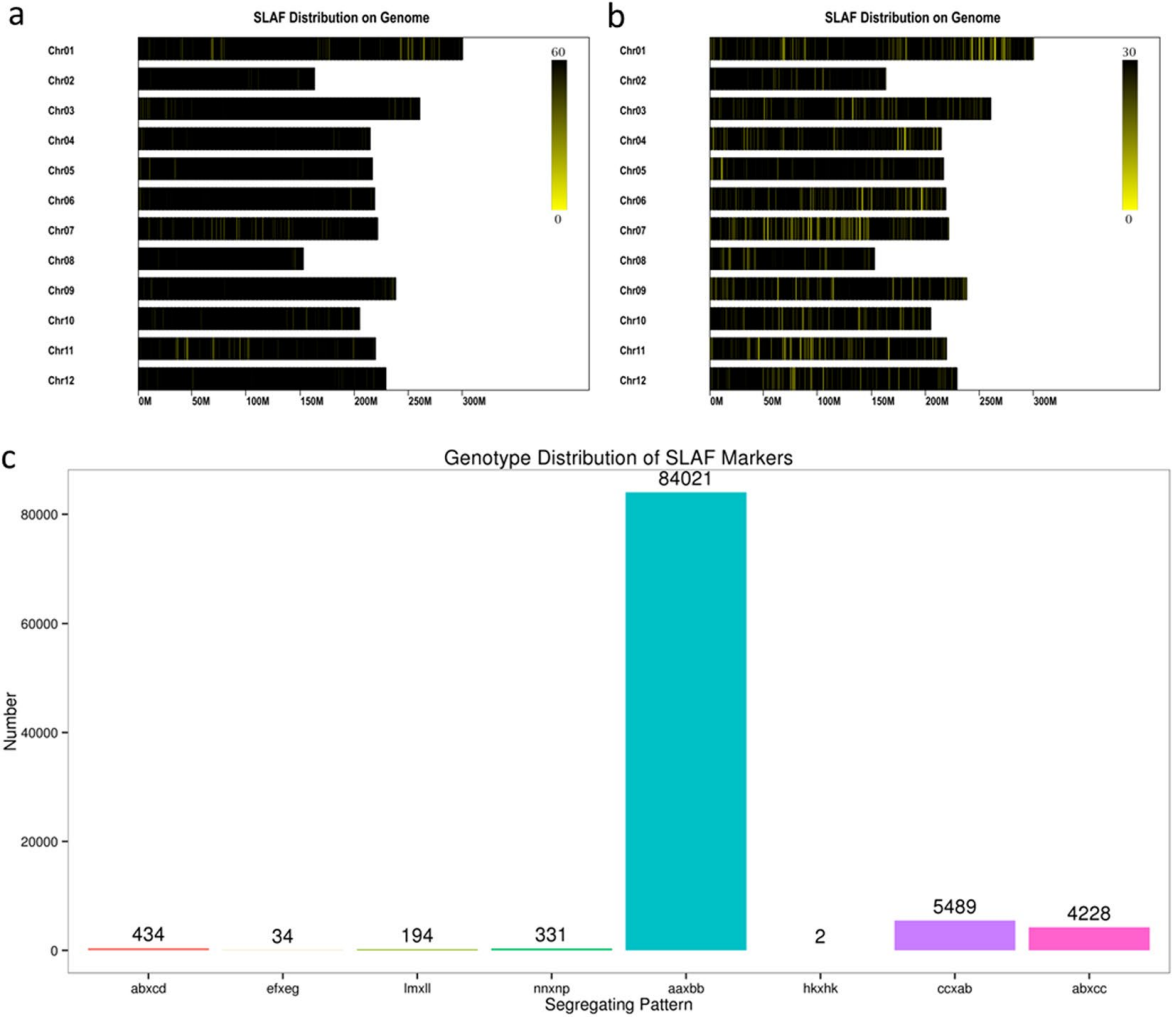
SLAF polymorphism analysis. (**a**) All SLAFs (black lines) distributed on 12 chromosomes. (**b**) Polymorphic markers distributed on 12 chromosomes. (**c**) Number of markers for eight segregation patterns. The x-axis indicates eight segregation patterns of polymorphic SLAF markers; the y-axis indicates the number of markers. F_2_ population is obtained from a cross of two pepper inbred lines with the genotype aa or bb; therefore, only the SLAF markers, which had segregation patterns of aa × bb, were used in map construction.

Based on the results of SLAF positioning on the Zunla-1 (*Capsicum annuum*) genome, the SLAFs on each chromosome were calculated (Table 2), and a distribution diagram of SLAFs on each chromosome is shown in Fig. 1a,b. The SLAFs were distributed equally on each chromosome, and the pepper genome has been successfully simplified. Among the 406,563 high-quality SLAFs, 171,413 were polymorphic according to an analysis of allele numbers and the differences between gene sequences, with a polymorphism rate 42.16%. Of the 171,413 polymorphic SLAFs, 94,733 were classified into eight segregation patterns (ab × cd, ef × eg, hk × hk, lm × ll, nn × np, aa × bb, ab × cc, and cc × ab) (Fig. 1c). Because the F_2_ population was obtained from a cross of two diverse pepper inbred lines with the genotype aa or bb, only the SLAF markers that had segregation patterns of aa × bb were used in map construction.

**Table 2:** Basic characteristics of pepper 12 linkage groups.

LG ID	SLAF	Polymorphic	Marker Number (%)	Total Distance	Average	Gaps < = 5	Max Gap	SNP
Chr1	38,266	16,656	123 (0.73)	121.41	1	95.90%	15.77	374
Chr2	21,986	9,965	1088 (10.91)	83.6	0.08	100%	1.6	3,327
Chr3	35,243	15,042	1730 (11.50)	188.3	0.11	99.71%	16.11	5,565
Chr4	29,135	11,594	285 (2.45)	142.36	0.5	98.94%	12.14	869
Chr5	29,925	13,646	751 (5.50)	181.78	0.24	99.60%	17.2	2,368
Chr6	30,193	11,869	1355 (11.41)	75.14	0.06	100%	2.75	4,412
Chr7	28,363	11,451	1107 (9.66)	114.92	0.1	99.82%	9.11	3,657
Chr8	22,002	9,026	203 (2.24)	151.98	0.75	99.50%	5.29	488
Chr9	33,315	13,915	401 (2.88)	88.41	0.22	99.75%	13.3	1,290
Chr10	27,060	11,939	375 (3.14)	143.3	0.38	99.20%	14.41	1,191
Chr11	27,123	12,271	922 (7.51)	160.42	0.17	99.78%	15.89	3,037
Chr12	31,092	13,187	698 (5.29)	135.16	0.19	99.71%	6.3	2,163
Total	406,563	171,413	9,038 (5.27)	1,586.78	0.18	99.33%	17.2	28,741

The % in the Marker Number column indicates the percentage of polymorphic SLAFs used for map construction.

### High-density genetic map constructed with SLAF markers

**Figure 2:**
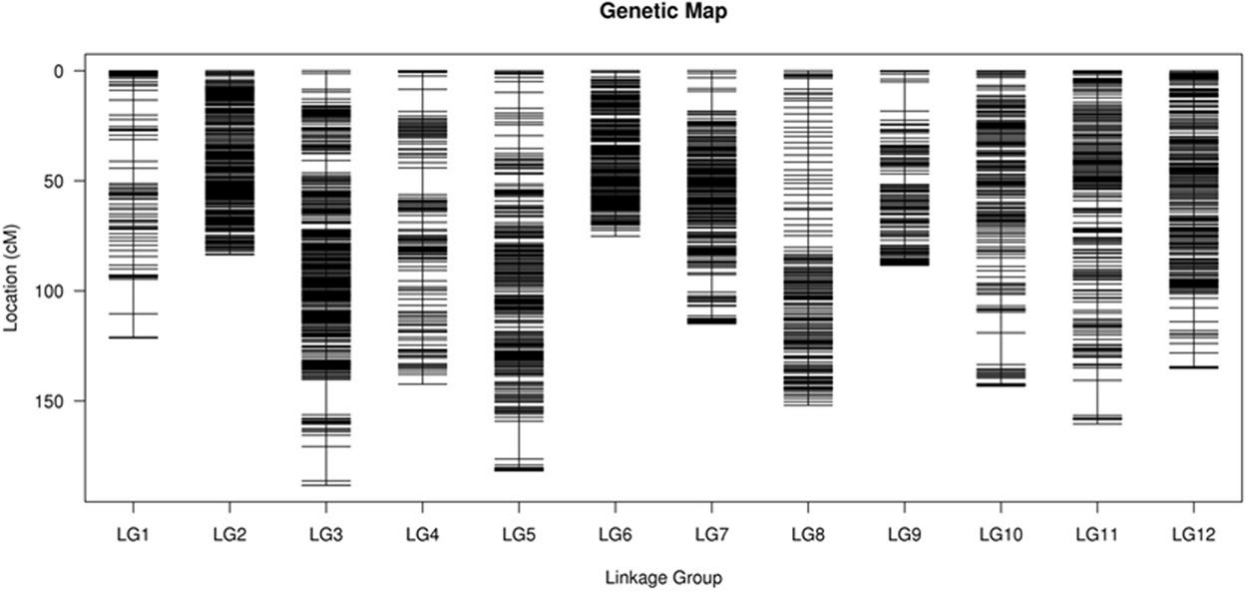
Distribution of SLAF markers on 12 linkage groups of pepper. A black bar indicates a SLAF marker. The x-axis represents the linkage group number, and the y-axis indicates the genetic distance.

To ensure the accuracy of genotyping, the following steps were performed as described during processing: (1) SLAFs with depth of less than 18-fold in each parent were discarded, (2) SLAFs with more than five SNPs were removed, (3) SLAFs with distorted segregation ratios (*χ*^2^ test, *p* < 0.05) were also maintained for genetic map construction, and (4) the markers demonstrating less than 85% integrity were discarded. Finally, 13,472 high-quality makers were distributed into 12 chromosomes according to their physical locations on the pepper reference genome and the MLOD scores >3 with other markers. As a result, 9,038 markers were designated for use in the final linkage map construction (Supplementary Tables 1 and 2). These markers are homozygous in the two parents, with a sequence depth 150-fold for the male parent and 101.4-fold for the female parent, respectively, and have more than 99% integrity of SLAFs for individuals. The polymorphic SLAFs used for the map construction ranged from 0.73% to 11.50% among the 12 linkage groups (LGs) (Table 2). Finally, the map contained 12 LGs and spanned a genetic distance of 1,586.78 cM in total, with an average distance of 0.18 cM between adjacent markers (Table 2). The distribution of the SLAF markers on each LG is shown in Fig. 2. On average, each LG contained 753 markers that covered an average of 132.23 cM. As shown in Table 2, the largest LG was Chr2, and it spanned a length of 188.3 cM, with 1,730 markers and an average distance of 0.11 cM between adjacent markers. In contrast, the smallest LG was Chr6, which harbored 1,355 makers, covered a length of 75.14 cM, and had a 0.06 cM average intermarker distance. The largest gap of 12 LGs ranged from 1.6 (Chr2) to 17.2 cM (Chr5). This genetic map included 28,741 SNPs (Table 2).

### Evaluation of the *Capsicum* Genetic Map

The quality of the genetic map was evaluated by using HighMap to construct haplotype map and heat map. The haplotype maps, which reflect the double recombination and deletion of the population, were generated for the parental controls and 150 offspring using 9,038 SLAF markers. In this study, most of the recombination blocks were distinctly defined. The missing data for each LG ranged from 0.76% to 1.14% (Supplementary Table 3). Most of the LGs were uniformly distributed, suggesting that the genetic maps were of high quality. The heat maps showed the relationships of the recombination between markers from each LG. The comparisons between markers were used to assign recombination scores to the 9,038 SLAF markers, after which the heat maps were constructed. The resulting maps showed that the order of the SLAF markers in most of the LGs have been correctly ordered (Supplementary Fig. S1). In total, 803 distorted SLAF markers were integrated into the map (Supplementary Table 4). They were noted in all LGs except Chr2 and Chr3, and most of the distorted markers were skewed toward the male parent. The frequencies of the distorted markers in Chr4 and Chr12 were much higher than those of the other LGs.

**Figure 3:**
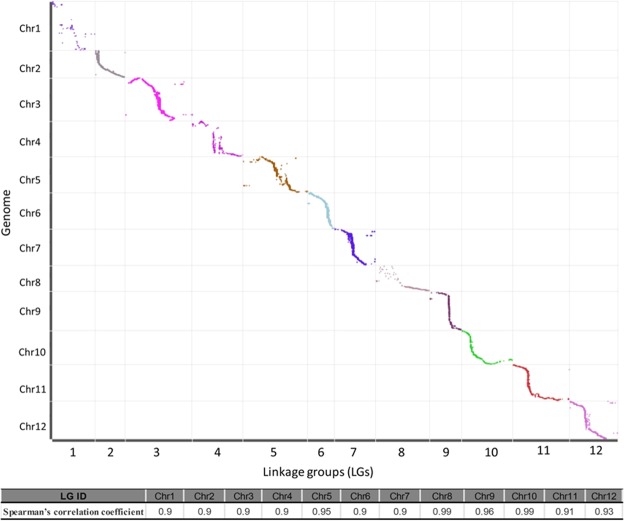
Collinearity of 12 chromosomes with the *Capsicum* reference genome. The x-axis indicates the genetic distance of the *Capsicum* chromosomes, and the y-axis represents the linear order of the physical position in the *Capsicum* genome. All 9038 SLAF markers in these chromosomes are plotted as dots on the figure. Different colors indicate different chromosomes. Spearman’s correlation coefficient of the genetic map and the physical map was presented in the bottom of the figure.

To evaluate the collinearity between the genetic map and the Zunla-1 reference genome, all SLAF markers were anchored on the *Capsicum* reference genome. As the results presented in Fig. 3, a sufficient genome coverage and the accurate genetic location of the markers was revealed by the consecutive curves except for Chr1 and Chr8. Nevertheless, the Spearman rank correlation test of the genetic map and the physical map revealed that the correlations were significant (*p* < 0.001) among the 12 chromosomes, indicating that the 9,038 SLAF markers were accurately positioned on 12 chromosomes, and the *Capsicum* genome was sufficiently covered with these SLAF markers. The genetic arrangements of most markers were also considered to coincide with their physical direction based on the falling trend of the curve.

### QTL analysis using the high density genetic map

**Figure 4:**
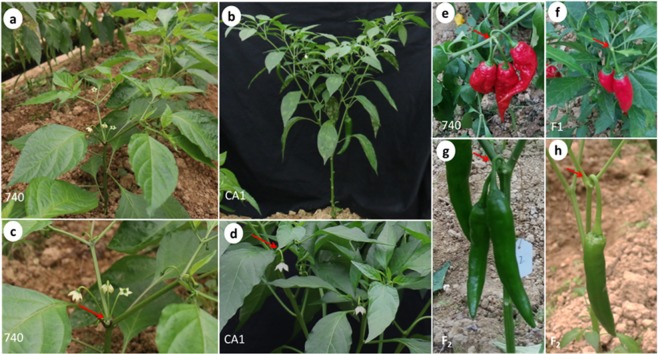
Phenotypes of the inbred lines 740 and CA1 and their F_1_ and F_2_ populations. (**a**,**b**) The 740 and CA1 plants in the flowering stage. (**c**,**d**) A node close-up of the 740 and CA1 plants, with the 740 plants showing multiple flowers per node and the CA1 plants showing one flower per node. (**e**,**f**) 740 and F_1_ individual fruits in the mature stage. (**g**,**h**) Pepper F_2_ individuals with two fruits (**g**) Or one fruit (**h**) Per branch node, respectively.

For floral phenotypic data collection, populations were planted in a greenhouse. As shown in Fig. 4, flowering time and flower number per node from the inbred line of 740 and CA1 displayed obviously different habits. For the inbred line of 740, the plants were with late flowering (flowering-score was 1), with multiple flower number per node (Fig. 4a,c; Supplementary Table 5 and 6). In contrast, the inbred line of the CA1 plants were early flowering (flowering-score was 6), with one flower per node (Fig. 4b,d; Supplementary Tables 5 and 6). As a result, the fruit in the parental and offspring populations mainly ranged from 1 to 3 per node (Fig. 4e–h).

Phenotypic data of flowering time and flower number per node of the two parents, and the F_1_ and F_2:3_ families were collected from three environments across three years. In all experiments, the phenotypic data flowering time of the two parents (740 and CA1) were located at the extreme ends of the largely normally distributed family means, and those for the F_1_ and F_2:3_ families were close to the mid-parent values, suggesting the quantitative genetics of these traits (Supplementary Fig. S2; Supplementary Tables 5 and 6). For the multiple flower trait, the CA1 parent averaged only one flower per node compared to 2.3 for 740. The F_1_ produced approximately 1.7 flowers per node, whereas the F_2:3_ were observed with the mean (1.5 flowers/node) being skewed toward the CA parent, suggesting incomplete dominance of the character. The coefficients of variation (CV%) of flowering time (47.79–50.01%) and multiple flowers (34.87–36.24%) in the F_2:3_ populations were significantly higher than the corresponding CV% from parents and F_1_, indicating the existence of real variation in our populations. Despite being collected over different years, data for these traits were highly consistent and of good quality, which provided a solid foundation for the QTL analysis.

### QTL analysis of flowering time and flower number per node traits

We conducted QTL analysis using the composite interval mapping (CIM) approach with data for each year in R/QTL. The LOD threshold for declaring significance of a QTL for flowering time and flower number per node was determined with 1000 permutations (*p* = 0.05). Details of each detected QTL, including the map location, LOD value, percentages of total phenotypic variances explained (*R*^*2*^), and 1.5-LOD support interval are presented in Table 3 and Supplementary Table 7, and the related SLAFs sequence are listed in Supplementary Table 1. As shown from the results, three flowering time QTLs, *Ft2*.*1*, *Ft6*.*1* and *Ft6*.*2*, and three flower number per node QTLs, *Mf2*.*1*, *Mf7*.*1* and *Mf10*.*1*, were consistently detected in all three environments. These QTLs could explain 46.35–50.37% and 99.13–99.76% of flowering time and flower number per node of the phenotypic variations, respectively. For the three flowering time QTLs, *Ft2*.*1* had the largest effect, which accounted for 24.59–27.87% of the phenotypic variations. With regard to the three flower number per node QTLs, *Mf2*.*1* had the largest effect, explaining approximately 40% of the phenotypic variations, and this was followed by *Mf7*.*1* and *Mf10*.*1*, which explained approximately 30% of the phenotypic variations. Of these QTLs for the detected traits, the interval ranged from 0.382 cM to 5.343 cM, and the peak LOD score ranged from 2.45 to 7.59. Combining the results from the QTL analysis with the QTLs for the three flowering times and the three flowers per node average explained 48.62% and 99.43% of the phenotypic variations across the three years, respectively. To obtain a more reliable QTL analysis, genome-wide composite interval mapping (GCIM) was performed with the QTL.gCIMapping.GUI package under a random model. QTLs were identified on Chr2, Chr 6, Chr7, Chr8, Chr10 and Chr11. For the flowering time traits, the QTLs detected with the CIM method were also identified by the GCIM method at least in two environments. The GCIM detected QTL peak LOD scores ranging from 5.16 to 22.7, which were higher than the QTL peak LOD score ranging from 2.45 to 5.43 and detected by the CIM method, and the detected QTL interval with the GCIM method was narrower than the corresponding QTL identified by the CIM method. The results for these QTLs explained 4.96–33.5% of the observed phenotypic variation phenotypic variations. For the flower number per node, the QTLs detected by the CIM method were consistently detected by the GCIM method. The QTLs peak LOD score detected by the GCIM were higher than the corresponding QTLs detected by the CIM, while the QTLs explained the observed phenotypic variation phenotypic variations were decreased. Nevertheless, two novel minor QTLs (i.e., *Mf8*.*1*, *Mf11*.*1*) were also detected by the GCIM method in at least two environments. The results for these QTLs explained 5.84–38.16% of the observed phenotypic variation phenotypic variations. These QTLs could explain 29.99–60.62% and 72.47–82.52% of flowering time and flower number per node of the phenotypic variations, respectively.

**Table 3:** QTL associated with pepper flowering time in pepper F_2:3_ families across three years.

Detection method	Year	QTL	Chr	Marker interval	Interval (cM)	Associate marker (cM)	Peak LOD	*R*^2^ (%)
CIM method with R/QTL	2014	*Ft2*.*1*	2	Marker6530388-Marker6433087	0.763	Marker6371227 (11.92)	5.12	25.52
	2014	*Ft6*.*1*	6	Marker3582600-Marker3748750	1.145	Marker3533009 (5.421)	2.96	12.18
	2014	*Ft6*.*2*	6	Marker3574378-Marker370912	3.435	Marker3515673 (9.620)	2.95	11.14
	2015	*Ft2*.*1*	2	Marker6530388-Marker6448176	1.145	Marker6207641 (11.611)	5.43	27.87
	2015	*Ft6*.*1*	6	Marker3566196-Marker3484733	2.672	Marker3634965 (5.803)	3.21	13.57
	2015	*Ft6*.*2*	6	Marker3784653-Marker343103	2.672	Marker3985111 (11.147)	2.48	9.23
	2017	*Ft2*.*1*	2	Marker6371227-Marker6448176	0.764	Marker6433087 (12.374)	4.92	24.59
	2017	*Ft6*.*1*	6	Marker3893018-Marker3748750	1.527	Marker3809982 (4.658)	2.45	10.53
	2017	*Ft6*.*2*	6	Marker3355152-Marker396246	5.343	Marker3902596 (12.292)	2.6	11.23
GCIM method with QTL.gCIMapping	2014	*Ft2*.*1*	2	Marker6371227-Marker6371227	—	Marker6371227 (11.92)	22.72	33.50
	2014	*Ft6*.*1*	6	Marker3686000-Marker3360006	0.763	Marker3360006 (6.50)	15.3	17.13
	2014	*Ft6*.*2*	6	Marker3784653-Marker3727891	0.382	Marker3727891 (11.4)	9.19	9.99
	2015	*Ft2*.*1*	2	Marker6448176-Marker6448176	—	Marker6271583(14.6)	20.49	28.76
	2015	*Ft6*.*1*	6	Marker3566196-Marker3566196	2.672	Marker3634965 (5.8)	6.42	4.96
	2015	*Ft6*.*2*	6	Marker3784653-Marker343103	2.672	Marker3709125 (12.67)	8.01	6.07
	2017	*Ft2*.*1*	2	Marker6433087-Marker6433087	—	Marker6433087 (12.43)	12.7	23.01
	2017	*Ft6*.*2*	6	Marker3857068- Marker3857068	—	Marker3857068 (11.15)	5.16	6.98

### Prediction of gene control flowering time and multiple flower trait

**Figure 5:**
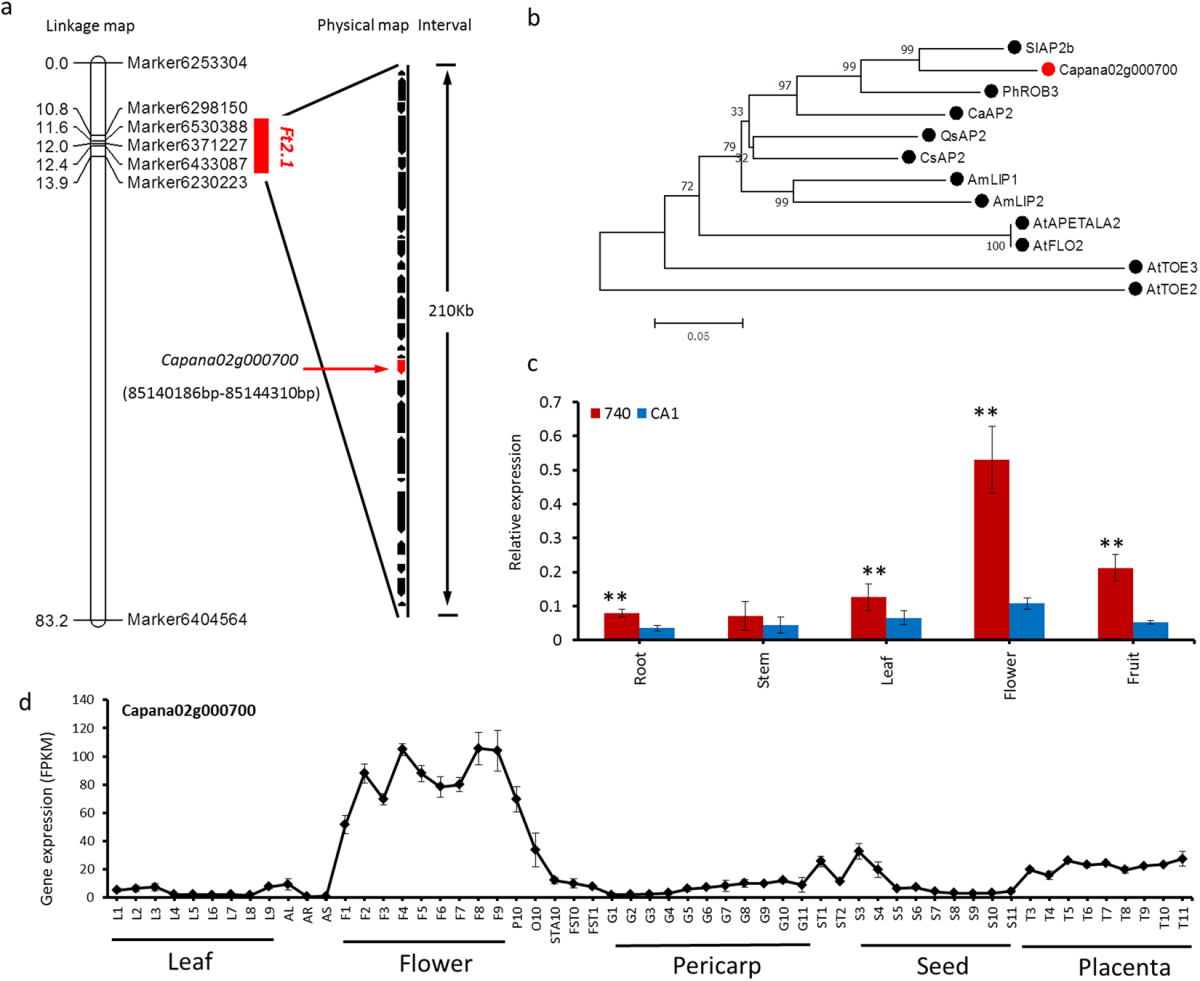
Local genetic linkage map showing the major QTL and identification of a floral homeotic protein, APETALA2, located in the major QTL region. (**a**) The major QTL for flowering time mapped to the interval between markers Marker6530388 and Marker6433087 on chromosome 2. The black arrow indicates the predicted genes in the interval, and the red arrow indicates the candidate gene *Capana02g000700*, which is associated with flowering time. (**b**) Phylogenetic tree of Capana02g000700 and its homologs. Numbers indicate the percentage of bootstrap support for each branch (1000 replicates). PhROB3 (APG29274.1), SlAP2b (NP_001233908.2), AmLIP1 (AAO52746.1), AmLIP2 (AAO52747.1), CsAP2 (AFK29251.1), QsAP2 (XP_023883493.1), AtTOE3 (NP_201519.1), AtTOE2 (NP_001189625.1), AtAPETALA2 (NP_195410.1), AtFOL2 (OAO99308.1), and CaAP2 (AJC11181.1). (**c**) qRT-PCR analysis of *Capana02g000700* expression in different 740 and CA1 tissues. Data presented are mean values of three biological repeats with three biological replicates s.d. ^**^P < 0.01 (Student’s *t*-test). (**d**) Digital gene expression level of *Capana02g000700* presented as FPKM values in the inbred line 6421 (*C*. *annuum*) from different tissues at different developmental stages. AL, all leaves; AR, all root; AS, all stem; P10, fully blossomed flower petals; O10, ovary with stigma; STA10, stamen.

To test the accuracy and precision of our genetic map, given that both the flower time *Ft2*.*1* and flower number per node *Mf2*.*1* QTL are the most reliable and can explain most phenotypic variation among the detected QTLs, the QTLs underlying the control of flowering time *Ft2*.*1* and flower number per node *Mf2*.*1* were used for gene prediction. For the *Ft2*.*1*, QTL region was mapped to 0.763 cM, and the interval physically represents approximately 210 kb in the Zunla-1 reference genome, with 25 putative predicted genes being included (Fig. 5a; Supplementary Table 8). Strikingly, *Capana02g000700* (85140186bp-85144310bp on chromosome 2), which encodes a floral homeotic protein, APETALA 2, was identified. Phylogenetic analysis indicated that the *Capana02g000700* homologs from *Antirrhinum* LIP1 and LIP2^25,26^, *Arabidopsis thaliana* APETALA2^27^, and pepper *CaAP2*^28^ play an important role in floral development (Fig. 5b). Analysis of the nucleotide sequence of *Capana02g000700*, 8 SNPs and a 6 bp deletion were detected among the 740 and CA1 ORF regions (Supplementary Fig. S3), which lead to predicting that the protein from CA1 has two amino acid deletions and five amino acid changes, including an amino acid in the AP2 domain (Supplementary Fig. S4). The qRT-PCR analysis of *Capana02g000700* expression indicated the expression was significantly more upregulated in the floral than other detected tissues (Fig. 5c). A comparison of the 740 and CA1 mRNA amount in the different tissues indicates its expression in 740 was significantly higher than in the corresponding CA1 (Fig. 5c). The RNA-Seq expression data in flower, leaf and fruit differential developmental stages were retrieved from the pepper inbred line 6421 (*C*. *annuum*)^29^, and *Capana02g000700* was found to have a higher expression level in pepper flower than in other tissues (Fig. 5d), which indicates that it plays an important role in flower development, strongly suggesting that *Capana02g000700* is candidate gene for transcriptional repression in the control of flowering time of pepper.

With regard to the *Mf2*.*1*, the QTL region was mapped to 0.382 cM, and the interval physically represents approximately 1400 kb in the Zunla-1 reference genome, and 98 putative predicted genes were found to reside in this region (Supplementary Fig. S5a; Supplementary Table 9). Inflorescence architecture is based on changes in the activity of the meristems, small groups of stem cells located at the tips of shoots^30,31^. Studies illustrated that, during the vegetative transition to flowering, the dynamically expressed genes were found enriched for transcription factors^32^. In the *Mf2*.*1* region, GO and COG analysis indicate that the genes involved in the transcription regulation process were enriched (Supplementary Fig. S5b). We retrieved the pepper transcriptome data of the middle vegetative meristem (MVM), transition meristem (TM), and floral meristem (FM) tissues reported previously^32^. Expression analysis revealed that a large number of genes were upregulated in TM, including TF from different family such as GATA (*Capana02g002708*, *Capana02g002714*), NAC (*Capana02g002682*), Zinc-finger homeodomain protein (*Capana02g002717*), and bHLH (*Capana02g002736*) (Supplementary Fig. S5c). These upregulated TFs within the *Mf2*.*1* region may be the candidates involved in control of transition meristem to form of floral meristem and then determine flower numbers.

### Coexpression analysis of genes expression

**Figure 6:**
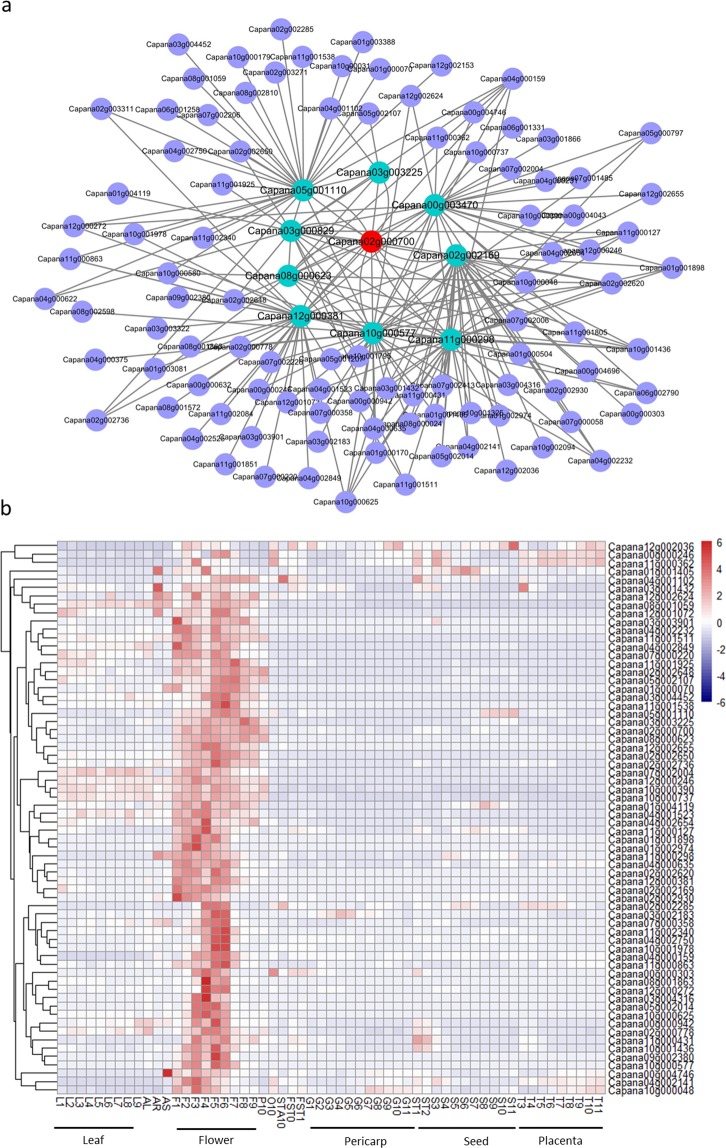
Co-expression analysis genes associated with flower development in pepper. (**a**) Co-expression analysis of genes co-expressed with *Capana02g000700*. The light green colour indicates the genes that are most highly co-expressed with Capana02g000700. (**b**) Heat map displaying 65 expressed genes selected from (**a**) pepper in differential development stages. AL, all leaves; AR, all roost; AS, all stems; P10, fully blossomed flower petals; O10, ovary with stigma; STA10, stamen.

We adopt the WGCNA to identify genes with differential expression at distinct stages of flower development. We identified a flower-development-specific module, which contained 107 genes (Fig. 6a; Supplementary Table 10). Of these genes, ten transcription factors (TFs), including *Capana02g000700*, were identified, indicating that these TFs may be involved in the transcriptional control of flower development. Strikingly, among these genes, nine were found highly co-expressed with *Capana02g000700*, and three TF *Capana11g000298* (bZIP family), *Capana08g000623* (MADS-box family), *Capana05g001110* (bHLH family) were detected as coming from different families (Fig. 6a). *Capana08g000623*, an orthologous gene from *A*. *thaliana PISTILLATA*, was reported as being involved in flower development and its expression regulated by the AtAPETALA2^33^. In tobacco plants, reduced amounts of *TGA2*.*1* (*Capana11g000298* orthologous gene) from tobacco resulted in the development of petal-like stamens, indicating a regulatory role of TGA2.1 in defining organ identity in tobacco flowers^34^. In addition, *Capana05g001110* encoding brassinosteroid enhanced expression 3 (BEE3) homologue positively regulated brassinosteroid signaling and required flower normal growth in *Arabidopsis*^35^. A heat map shows the expression (FPKM) of 65 genes selected from Fig. 6a and found most of the expression of these genes was flower-development regulated (Fig. 6b). These results strongly support that *Capana02g000700* may repress target genes and/or TFs expression to regulate flower development and then affect flowering time.

## Discussion

### High-density genetic map constructed with *C*. *chinense* and *C*. *annuum* interspecies hybridization F_2_ population

The pepper genome sequence has been completed, and the size of the pepper genome is large, estimated to be 3.48 Gb^3,36^. Whole-genome deep resequencing or low-coverage sequencing is relatively costly for large genomes and usually unnecessary for gene/QTL mapping^11,37^. The SLAF-seq, which was developed based on high-throughput sequencing, is an effective strategy for large-scale SNP discovery and genotyping^20^. In contrast to conventional methods, which are inefficient, expensive, and time-consuming^20,38^, SLAF sequencing can generate large amounts of sequence information and handle whole genome density distributions, which ensures density, uniformity, and efficiency of marker development^20,39,40^. In this study, we constructed a high-density genetic map using the SLAF-seq technology with an interspecies F_2_ population consisting of 150 individuals. This map, which included 9,038 high-quality SLAF markers on 12 LGs, covered a genetic distance of 1,586.78 cM in total, with an average distance of 0.18 cM between adjacent markers, and showed 99% integrity for individuals (Fig. 3, Table 2). To date, compared with other genetic maps of *Capsicum* species^16,24,41,42^, the genetic map reported in this study is the highest density map and had the smallest average distance between interval markers for *Capsicum* genus plants. With this high-density map combination with high-quality genome sequences, the candidate genes within a narrow region interval between adjacent markers can be predicted directly. However, as reflect in our results we also need to keep in mind that success QTL mapping depend on the chromosome region the QTL falls into. Overall, SLAF-seq technology is ideal for population genotyping and for high-resolution linkage map construction because of its high success rates, specificity, and stability^20^. Accordingly, the genetic map could be used for detecting QTL for important horticultural traits, and the narrowed QTLs also provided several promising candidate genes for further functional identification.

Segregation distortion is a commonly observed in both interspecific and intraspecific cross populations^43^. In this study, to avoid the losing information, the 803 SLAF markers that were also utilized in map construction. In total, almost all the segregation makers were skewed toward the male parent. The distorted segregation could be caused by gametophytic factors that affect female gametes^43,44^, but this distortion needs further study. We found that the rate of the polymorphic SLAFs in the Chr1 and Chr8 being used for map construction were significantly lower than other LGs. Possibly, chromosome translocation between chromosome 1 and chromosome 8 in the inbred pepper lines 740 and CA1 and the many markers had to be filtered before being used for genetic map construction. Indeed, the translocation between chromosome 1 and chromosome 8 in *C*. *frutescens*, *C*. *chinense*, *C*. *baccatum* and wild *C*. *annuum* compared to cultivated *C*. *annuum* was previously reported and has been well characterized^45^. In addition, only *C*. *annuum-*originated sequences were used for developing the SLAF markers, whereas some *C*. *chinense* specific markers were unable to be detected. Therefore, the alignment of the SLAFs to the *C*. *chinense* genome needs to be developed for a more complete genetic map in the future.

### QTL identification of floral-related traits

The yield-related traits are important for pepper production, and high yield and high disease resistance always are the most important objectives for pepper breeding. Therefore, the detection of QTLs or genes for these traits should be important for pepper genetic improvement. Hitherto, QTLs studies on yield related traits have been widely documented in intraspecific and interspecific crosses of *Capsicum* species populations^8,12,46,47^. Previous studies have strongly indicated that selection for early flowering can enhance yields in various horticultural types of pepper. Flowering time is a fully quantitative complex trait, and researchers always record the number of days between sowing and anthesis or at certain day after sowing to score the flower or fruit developmental status of the third node to evaluate flowering times^8,15^. In addition, some others also used the leaves numbers on primary stem to evaluate flowering time^28^. After comprehensively comparing the characteristic of parents and progenies used in this and previous studies, we selected the score criterion for evaluating the flowering time, and three QTLs were detected in the study (Table 3). For flowering time, the major QTL *Ft2*.*1* explains approximately 30% of the phenotypic variation detected on Chr2 in our populations across the three years. The major QTL related to flowering time in the Chr2 was also detected by using the RILs derived from the interspecific cross of the *C*. *frutescens* × *C*. *annuum*^15^. A comparison of the physical position reported previously, adjacent to the *Ft2*.*1* QTL interval, revealed a gene previously reported and mapped on the Chr2 from different studies^14,28^, which promotes the phase transition from inflorescence meristem to floral specification in pepper *CaAP2* (*Capana02g003062*). In addition, in the *C*. *annuum* intraspecific F_2_ populations, using an SLAF-seq and BSA analysis, a candidate region was mapped on Chr12 that controls the first flower node and determines the flowering time^47^. However, the *Ft2*.*1* not located in a previously reported physical position, indicated the *Ft2*.*1* is a new locus that plays an important function in controlling flower development in the *Capsicum* genus among plants of different genetic backgrounds. In addition, among the two QTLs detected on Chr6, the novel QTLs *Ft6*.*1* and *Ft6*.*2* detected in this study differ from previous studies, suggesting that perhaps the population used for QTL identification came from different species, as previously reported. However, the three detected QTLs, which accounted for approximately of 50% of the total phenotypic variance, were detected with two methods, which may have been because the flowering time was scored based on a visual scale of 1–6, which limits the resolution of mapping a fully quantitative trait. Alternatively, markers with significant segregation distortion were used for genetic map construction, and these distorted markers may have affected the QTL analysis^43,44,48^. A more detailed scoring criterion may contribute to detecting more QTLs for the flowering time.

Possibly, the gene for the multiple flower trait could be transferred from the *C*. *chinense* to *C*. *annuum* varieties with a more concentrated fruit set, thereby contributing to a potentially higher yield^10^. In this study, we found the hybrid progenies with multiple flowers plants can produce an average of approximately 1.5 flowers per node (Supplementary Table 6). In addition, we observed that progeny with multiple flowers are always accompanied with more than one fruit setting in a node (Fig. 4), and this result was consistent with results reported previously by others^9,10^. Previous studies have proposed hypotheses for different genetic control mechanisms of multiple flowers: (i) a three-gene dominance model with epistasis^49^, (ii) at least five independently segregating chromosome segments involved in the multiple-flower habit^9^, (iii) seven semi-recessive additive genes from *C*. *chinense*^50^, and (iv) three major dominant genes from *C*. *chinense*^10^. Possibly, the populations were delivered from differential accessions of *C*. *annuum* and *C*. *chinense*, and the interrelationships among these models are unclear. In this study, with the CIM and GCIM QTL analysis, the detected QTLs *Mf2*.*1*, *Mf7*.*1* and *Mf10*.*1*, with a positive additive effect (increased flower number), could exhibit greater phenotypic variation of the multiple flower trait. However, the GCIM method was also able to detect another two QTLs *Mf8*.*1* and *Mf11*.*1*. In addition, the QTLs detected by GCIM had higher LOD peak scores, had more narrowed interval and were greater in number, indicating that GCIM mapping approach is more effective and reliable for detect more QTLs. Over all, the result seems consistent with the hypothesis of five-gene model of genetic control of multiple flowers in *Capsicum*^9,10^, but this needs further study. Nevertheless, we provided more detailed loci information, and this could contribute to uncovering the underlying genetic and molecular mechanism of multiple flowers traits in *C*. *chinense*. In addition, three QTLs related to multiple flowers *Mf2*.*1*, *Mf7*.*1*, *Mf8*.*1*, *Mf10*.*1* and *Mf11*.*1* were mapped in a narrow interval, which means that molecular markers closely linked to multiple-flower-per-node locus can be more effectively applied in early selection. Within the *Mf2*.*1* region, even though we identified many TFs and found the expression of these TFs were upregulated in the transition meristem, these TFs may play an important role in control of vegetative meristem transition to floral meristem, which determines inflorescence architectures (flower number determinants). However, we found *ANANTHA* (*AN*) and *COMPOUND INFLORESCENCE* (*S*, the homolog of WUSCHEL-RELATED HOMEOBOX 9, WOX9) and their orthologs genes have conserved functions in the control of inflorescence architecture among Solanaceae species^31,51^. Given that the QTL can vary according to various factors such as mapping populations, genotyping method, detection method and environmental factor^43,44,52^. When considering these factors, we cannot rule out the possibility that these genes were involved in the control of the trait of flower number per node between *C*. *annuum* and *C*. *chinense* species. Further study is needed to finely map and identify the candidate genes underlying molecular mechanism control of multiple flowers and for the development of reliable makers for marker-assisted selection to pyramid the genes that control multiple-flower into commercial cultivars.

### Multiple strategies to prediction of flowering time candidate genes

Within the flower major region *Ft2*.*1*, we were able to identify a candidate gene for the regulation of flowering time and flower development. After annotation of the 25 genes resides in genomic regions, we found *Capana02g000700* encoding a floral APETALA2 protein, whose homologs from AtAPETALA2 and AtFLO2 act as flowering suppressors (Fig. 5b). In addition, we found *Capana02g000700* expression significantly upregulated in flowers, and we also determined its expression in late flowering 740, where the expression was significantly higher than observed for the early flowering CA1 (Fig. 5c). Furthermore, we found many flower developmental-stage-specific genes were highly coexpressed with *Capana02g000700* (Fig. 6). In a sequence comparison of CDS between the parents used for the QTL mapping, we detected 8 SNPs and a 6 bp deletion in CA1, but none of these changes result in a premature stop codon (Supplementary Figs S3, S4). We cannot rule out the possibility that the sequence variations between 740 and CA1 may have caused change in the gene activity. However, after comparing the *Capana02g000700* gene from the *C*. *annuum* and *C*. *chinense* reference genomes reported before, we found the SNPs were highly conserved among the same species (Supplementary Figs S3 and S4), and we assumed that no major changes in the activity of the protein could be expected. In *Arabidopsis*, one of actions of AtAPETALA2 was to control flower development by repressing the flowering-promoter MADS-box transcription factor such as *PISTILLATA*^33^, *AGAMOUS*^40^ and *SOC1*^41^. So, the results presented in this study show that the higher expression of *Capana02g000700* in late flowering 740 results in enhanced repression of target flowering-promoter genes expression, which finally leads to late flowering. Although we could illustrate significant expression differences between the early flowering and late flowering parents, the underlying specific sequence variation in the promoter region associated with this difference is still unknown. We retrieved the *C*. *chinense* and *C*. *annuum* genome sequence reported previously^3,36,53^, and we found that the nucleotide sequence within the *Ft2*.*1* region was misassembled or that the assembly quality was quite low for many of the gaps presented. We tried to elucidate the DNA variations between CA1 and 740, but we failed to amplify of *Capana02g000700* promoter using the primers designed for the *C*. *annuum* genome sequence (Zunla-1 and CM334)^3,36^, indicating that a larger transposon may exist in its promoter. Because the gene detected in this study was different from the previously reported *CaAP2*, we assume the *Capana02g000700* detected in this study is *CaAP2* and *Capana02g000700*, which evolved from the same ancestor gene but evolved after the two AP2/ERFs (i.e., *CaAP2* and *Capana02g000700*). These genes, which evolved independently, control flowering time in a different genetic background. We hypothesized that the *Capana02g000700* mainly exerts control on the flowering time between *C*. *annuum* and *C*. *chinense* species, but further research is needed.

## Materials and Methods

### Plant materials

To develop a F_2_ population of interspecific hybridization between *C*. *chinense* and *C*. *annuum*, a cross was made between the inbred lines 740 (*C*. *chinense*) and CA1 (*C*. *annuum*). The female parent 740 plants multiple flowers per node and late flowering. In contrast, the plants of male parent CA1 has one flower per node and early flowering. The two parental lines were planted in greenhouse in the spring of 2013, the F_1_ of 740 × CA1 were grown in autumn of 2013 and self-pollinated to obtain 150 F_2_ and the F_2_ population were planted in the spring of 2014 and self-pollinated to obtain 150 F_2:3_ families.

### Phenotypic data collection

Phenotypic data were collected in three environments over 3 years. The parents, F_1_ and F_2:3_ (2014 autumn, 2015, 2017 spring) families were grown in green house or College of Horticulture at Guangzhou, China. The 740, CA1, F_1_ and F_2:3_ families were designed with two replicate, and planting with 8 plants in each replication. Flowering time-scores of one to six were given based on the developmental stage of the flower/fruit at the third node on day 90, and the scoring of individuals was according to previous reported method with little modifications^8^. The scores represented the following traits: 1 = no obvious flower bud, 2 = flower bud/flower, 3 = small fruits, 4 = small- to medium-size fruits, 5 = medium-size fruits and 6 = mature fruits. With respected to multiple flowers traits, the survey was completed after the flower number at the fifth node could be clearly distinguished, and plants were evaluated the numbers of the flowers produced per node for the first five sets of nodes. We measured the traits on individual plants, and averaged within each F_3_ family.

### DNA extraction

DNA was extracted from young health leaves of two parents and the 150 F_2_ offsprings by the method of CTAB^54^. The DNA samples were quantified with NanoDrop ND-1000 spectrophotometer (Wilmington, USA) and by electrophoresis in a 1% agarose gels with lambda DNA as a standard.

### SLAF library construction and high-throughput sequencing

The SLAF-seq strategy was used to analyse the genotype of two parents and 150 F_2_ offspring as described previously^20^. Briefly, the genomic DNA of the two parents and 150 F_2_ populations was digested using the *HaeIII* restriction enzyme (New England Biolabs, USA). Subsequently, a signal nucleotide overhang was added to the digested fragments along with Klenow fragments (New England Biolabs, USA) and dATP at 37 °C. Then, PAGE-purified Duplex Tag-labelled sequencing adapters (Life Technologies, USA) were ligated to the A-tailed DNA with T4 DNA ligase (New England Biolabs, USA). After incubation, the reaction products were pooled and purified using a Quick Spin column (Qiagen, Germany). The purified products were electrophoresed on a 2% agarose gel, and fragments with sizes ranging from 414 to 514 bp were collected and purified using a gel extraction kit (QIAGEN, Germany). The purified product was sequenced on an Illumina HiSeq. 2500 system (Illumina, USA) according to the manufacturer’s instructions.

### Sequence data analysis and genotyping

The SLAF marker grouping and genotyping were performed using procedures as described previous^20^. Briefly, after filtering out the low-quality reads (quality score < 30e), the remaining cleaned SLAF pair-end reads were clustered based on sequence similarity as alignment with BLAT (-tilesize = 10 -stepsize = 5). Subsequently, pair-end clean reads were mapped onto the reference genome of *C*. *annuum*. var Zunla-1^36^ and reads with over 90% similarity sequences were grouped into one SLAF locus. Minor allele frequency evaluation was used to define alleles in each SLAF locus. *C*. *annuum and C*. *chinense* are diploid species, one locus could harbor at most four SLAF tags, locus containing more than four tags were filtered out as repetitive SLAFs, and those with two, three, and four tags were identified as polymorphic SLAFs. Then polymorphic SLAFs were classified into eight segregation patterns (aa × bb, ab × cc, ab × cd, cc × ab, ef × eg, hk × hk, lm × ll and nn × np). Because the F_2_ population is obtained from a cross of two diverse pepper inbred line with the genotype aa or bb, therefore only the SLAF markers which had segregation patterns of aa × bb were used in map construction.

### Sequence data analysis and genotyping

The SLAF marker grouping and genotyping were performed using procedures as described previous^20^.

Briefly, after filtering out the low-quality reads (quality score < 30e), the remaining cleaned SLAF pair-end reads were clustered based on sequence similarity as alignment with BLAT (-tilesize = 10 -stepsize = 5). Subsequently, pair-end clean reads were mapped onto the reference genome of *C*. *annuum*. var Zunla-1^36^ and reads with over 90% similarity sequences were grouped into one SLAF locus. Minor allele frequency evaluation was used to define alleles in each SLAF locus. *C*. *annuum and C*. *chinense* are diploid species, one locus could harbor at most four SLAF tags, locus containing more than four tags were filtered out as repetitive SLAFs, and those with two, three, and four tags were identified as polymorphic SLAFs. Then polymorphic SLAFs were classified into eight segregation patterns (aa × bb, ab × cc, ab × cd, cc × ab, ef × eg, hk × hk, lm × ll and nn × np). Because the F_2_ population is obtained from a cross of two diverse pepper inbred line with the genotype aa or bb, therefore only the SLAF markers which had segregation patterns of aa × bb were used in map construction.

### Genetic map construction

In order to ensure the quality of genetic map, high-quality SLAF markers for the genetic map construction were filtered by the following criterions: (1) SLAF makers with parents sequence depth less than 18 were filtered out; (2) a SLAF which had less than five SNPs and average depth of each sample above four, was defined as a high quality SLAF marker; (3) markers with complete less than 85% were filtered; (4) Since distortedly segregated markers are ubiquitous and would affect the mapping construction and QTL analysis^43,44,48^, partial distorted polymorphism markers showing significance (*p* < 0.05) were maintained to construct the map. Subsequently, by using the HighMap strategy, the SLAF markers were assigned into chromosomes based on the pepper genome, and 12 linkage groups (LGs) were obtained. The modified logarithm of odds (MLOD) value was calculated between two adjacent makers, the SLAFs with MLOD values less than three were excluded. In addition, using HighMap software to analyze the linear array of markers in each LG, and estimate the genetic distances between two adjacent markers.

### QTL/Gene mapping

Plant trait QTLs were identified by different methods. QTLs were detected by CIM methods with the R/QTL package methods using R/QTL v3.1.1^55^. The significance thresholds were determined using 1,000 permutations (*p* < 0.05). The results from the CIM analysis were used to construct the QTLs, and their positions were used in a default model. In addition, multilocus QTL mapping was performed by the software of QTL.gCIMapping.GUI (https://cran.r-project.org/web/packages/QTL.gCIMapping/index.html) according to the user manual^52,56^.

### Candidate gene selection and annotation

The predicted genes in the target QTL region were analysed according to the annotation of the pepper Zunla-1 and CM334 reference genomes^3,31^. The function of genes detected in the candidate region was manually confirmed using protein BLAST. In addition, GO enrichment and KEGG pathway analyses were performed with default settings. Multiple sequence alignments were performed with ClustalX, and a phylogenetic tree was calculated by the neighbour-joining method and bootstrap analysis with 1000 replicates via MEGA7 software^57^.

### qRT-PCR analysis candidate gene expression level

Floral buds, fruit at developmental stage of 16 day post anthesis, leaves at 20 day after emergence, stem and root were collected from 740 and CA1, respectively. Samples were ground into fine powder in liquid nitrogen, and then the RNA was isolated from all samples using HiPure Plant RNA Mini Kit (Magen, China). Subsequently, the RNA from each sample was used for the reverse transcription reaction using a HiScript Q RT SuperMix for qPCR reagent kit with gDNA wiper (Vazyme, China). Quantitative real-time PCR analysis was performed on a LightCycler 480 Real-Time PCR System according to the manufacturer’s instructions; the qPCR program was according to described previously^2^ and primers used for analysis were listed in Supplementary Table 11. The reported values represent the mean of three biological replicates.

### Candidate gene cloning and sequence analysis

When the candidate gene sequence was cloned, the total RNA was extracted from the bud of the CA1 and 740 by using a HiPure Plant RNA Mini Kit (Magen, China). The RNA from each sample was used for the reverse transcription reaction using an ImProm-II Reverse Transcription System (Promega, USA). The cDNA samples served as the template for amplification with the LA Taq DNA polymerase (TaKaRa, Japan) for the gene-specific marker (Supplementary Table 11), and the PCR products were cloned into a pMD19-T vector (TaKaRa, Japan). Positive clones were picked to culture for plasmid extraction and sequencing.

### Co-expression analysis of gene expression

Co-expression network of gene expression was constructed with the weighted gene coexpression network analysis (WGCNA) package^58^ using gene expression data of different tissue samples from different developmental stages^29^. The modules were obtained using the automatic network construction function blockwiseModules with default settings. The genes co-expressed network was visualized by the Cytoscape 3.0^59^.

## Supplementary information

Supplementary Table 9

Supplementary Table 8

Supplementary Table 1

Supplementary Table 2

## Data Availability

Sequence data accession number (SUB4758068). *Capana02g000700* sequence of inbred line CA1 accession number: 2165004; *Capana02g000700* sequence of inbred line 740 Accession Number: 2165018.
